# Supplementary material for: CAMISIM: simulating metagenomes and microbial communities
Source: Microbiome. 2019 Feb 8;7:17. doi: 10.1186/s40168-019-0633-6 (PMC6368784; doi:10.1186/s40168-019-0633-6)
Supplement: Supplementary file 1 — Supplementary information. Taxonomic profile based community design: BIOM format details, Reference database. De novo community design: Creation of the mapping file. Genome assembly metrics. Methods: iTol, Parameters, PICRUST, Configfile. OTU mapping file. (PDF 146 kb) [file 40168_2019_633_MOESM1_ESM.pdf]

Supplementary information

CAMISIM: Simulating metagenomes and microbial communities

Contents

1 Taxonomic profile based community design **2**

1.1 BIOM format details 2

1.2 Reference database 2

2 De novo community design **3**

2.1 Creation of the mapping file 3

3 Genome assembly metrics **4**

4 Methods **5**

4.1 iTol 5

4.2 Parameters 14

4.3 PICRUST 15

4.4 Config file 16

5 OTU mapping file **19**

1 Taxonomic profile based community design

1.1 BIOM format details

The input BIOM profiles for the "from profile" mode of CAMISIM are required to either have the lineage stored as the ID, or preferably the field "lineage" in the "metadata". The lineage should be greengenes formatted, namely semicolon separated strings x_0__scientific name; x_1__scientific name with x_i being the taxonomic rank descriptor - 's' for species,'g' for genus,'f' for family,'o' for order,'c' for class,'p' for phylum, 'k' for superkingdom - and a scientific name of the taxon.

1.2 Reference database

CAMISIM requires a reference database of available complete genomes, created by algorithm 1. Per default all genomes tagged as "Complete genome" from the RefSeq are taken into account, but arbitrary reference data bases, online or offline, can be provided.

For all genomes $g \in G_{in}$, their taxonomy is known and given as a list with 7 elements tax_g, corresponding to their taxonomic identifier on the seven main taxonomic ranks (*species, genus, family, order, class, phylum, superkingdom*).
For example, the well-known bacteria *E. coli* has the NCBI taxonomy ID 562, it's genus Escherichia has the ID 561 and it's taxonomy goes up to the superkingdom "bacteria" with the taxonomic ID 2, so finally *E. coli*'s list would be $[562, 561, 543, 91347, 1224, 1236, 2]$. Given these lists it is possible to create a set of available complete genomes per taxon G_t.

Let for example the genome of *E. coli* and the genome of *E. albertii* (taxonomic ID 208962) be part of G_{in}, then we have two available genomes on the rank of species and one for every rank above, since *E. coli* and *E. albertii* belong to the same genus. This results in the sets per taxon $G_{562} = \{562\}$, $G_{208962} = \{208962\}$, $G_{561} = \{562, 208962\}$ etc. We add all the taxa for which a set G_t exists to the collection of sets F.

Algorithm 1: *create-reference*(G_{in})

input : List of complete genomes G_{in}

output: Collection of sets F which contains sets of taxonomic IDs G_t for every taxon t which has at least one complete genome available

R = [species, genus, family, order, class, phylum, superkingdom]
$F = \varnothing$ **foreach** $t \in G_{in}$ **do**
    get list tax_t from NCBI
    foreach $i \in tax_t$ **do**
        G_t.append($tax_t[0]$)
        $F = F \cup G_t$
    end
end
return F

2 De novo community design

In addition to providing genomes, it is possible to artificially add strain variation to CAMISIM. These additional strain genomes are created with a novel version of sgEvolver. In its original implementation, the simulated strain evolution process did not model natural selection. The lack of modeled selection could then cause the Open Reading Frames (ORFs) of protein encoding genes to break. Using prodigal, these ORFs are now initially found within the genomes sampling collection and mutation rates lowered for these genomic locations: substitutions that would introduce a stop codon (assumed here to be TGA, TAA, or TAG) are rejected with a probability of 98%. Secondly, substitutions that would eliminate an existing in-frame stop codon are rejected with 100% probability. Artificial strains evolved from real genomes are added to the community genome collection until the difference between genomes_total and num_real_genomes has been reached.

2.1 Creation of the mapping file

Genomes used for the *de novo* mode of CAMISIM require a "novelty category" assigned to them. To assign a novelty category to a genome from the input genome collection, genomes from same taxon are searched for in

a reference collection created from draft and complete genomes from NCBI RefSeq (microbial part), NCBI whole bacterial genomes, NCBI draft bacterial genomes and the NCBI Human Microbiome Project are searched for. The search begins at the species rank and the continues for genus, family, order, class, phylum and superkingdom. If a genome is found, the the novelty category is set to the rank visited below. For example, if a reference genome of the same species exists for a query genome, the novelty is declared as `new_strain`. If no reference genome of the same species is found, but there is a reference genome for another species of the same genus, the novelty category is set to `new_species`, etc. OTU IDs in the mapping file are inferred by extracting small rRNAs from these genomes and clustering these with small rRNA reference databases. The exact algorithm is found in the supplement of the CAMI main paper.

3 Genome assembly metrics

Evaluation of the assemblies was performed using quast, or metaquast in the case of the evolutionary relatedness evaluation, for quast default parameters were chosen. For metaquast two options of –ambiguity-usage were used (one and all), namely allowing single contigs to only map to a single reference genome or all (both), to explain the difference between strain and consensus contigs.
The metrics which are shown for the plot, Genome fraction, NGA50 and number of contigs should in combination yield a good picture of the assembly quality, while the individual metrics might not draw the complete picture: Genome fraction just describes the percentage of the reference genome which is covered by at least one contig. So having a lot of (overlapping) contigs results in the Genome fraction to look relatively well. This is complemented by the number of contigs metric, which punishes a high number of contigs. A low number of contigs is not a good measure for assembly quality alone since it does not include the measure how long these contigs are. This is why we chose NGA50 as the final metric to show. NGA50 describes the length of the aligned contig with which 50% of the reference genome is covered, so it is basically the combination of the first two metrics: Few and long contigs are similarly bad as many short ones and both result in the NGA50 to decline.

4 Methods

4.1 iTol

The 191 iTol genomes from the itol Nexus tree (Table 4.1) were mapped to the NCBI RefSeq data base and genomes downloaded accordingly, sequences shorter than 450k bp were filtered and all genomes which did not have an exact matching complete genome, resulting in 152 genomes with their NCBI ID (Table 4.1)

iTol Nexus tree

```
#NEXUS

Begin trees; [Treefile created Wed May 25 12:58:12 2016]
Translate
1 Escherichia coli EDL933,
2 Escherichia coli O157:H7,
3 Escherichia coli O6,
4 Escherichia coli K12,
5 Shigella flexneri 2a 2457T,
6 Shigella flexneri 2a 301,
7 Salmonella enterica,
8 Salmonella typhi,
9 Salmonella typhimurium,
10 Yersinia pestis Medievalis,
11 Yersinia pestis KIM,
12 Yersinia pestis CO92,
13 Photorhabdus luminescens,
14 Blochmannia floridanus,
15 Wigglesworthia brevipalpis,
16 Buchnera aphidicola Bp,
17 Buchnera aphidicola APS,
18 Buchnera aphidicola Sg,
19 Pasteurella multocida,
20 Haemophilus influenzae,
21 Haemophilus ducreyi,
22 Vibrio vulnificus YJ016,
```

23 Vibrio vulnificus CMCP6,
24 Vibrio parahaemolyticus,
25 Vibrio cholerae,
26 Photobacterium profundum,
27 Shewanella oneidensis,
28 Pseudomonas putida,
29 Pseudomonas syringae,
30 Pseudomonas aeruginosa,
31 Xylella fastidiosa 700964,
32 Xylella fastidiosa 9a5c,
33 Xanthomonas axonopodis,
34 Xanthomonas campestris,
35 Coxiella burnetii,
36 Neisseria meningitidis A,
37 Neisseria meningitidis B,
38 Chromobacterium violaceum,
39 Bordetella pertussis,
40 Bordetella parapertussis,
41 Bordetella bronchiseptica,
42 Ralstonia solanacearum,
43 Nitrosomonas europaea,
44 Agrobacterium tumefaciens Cereon,
45 Agrobacterium tumefaciens WashU,
46 Rhizobium meliloti,
47 Brucella suis,
48 Brucella melitensis,
49 Rhizobium loti,
50 Rhodopseudomonas palustris,
51 Bradyrhizobium japonicum,
52 Caulobacter crescentus,
53 Wolbachia sp. wMel,
54 Rickettsia prowazekii,
55 Rickettsia conorii,
56 Helicobacter pylori J99,
57 Helicobacter pylori 26695,
58 Helicobacter hepaticus,
59 Wolinella succinogenes,
60 Campylobacter jejuni,

61 Desulfovibrio vulgaris,
62 Geobacter sulfurreducens,
63 Bdellovibrio bacteriovorus,
64 Acidobacterium capsulatum,
65 Solibacter usitatus,
66 Fusobacterium nucleatum,
67 Aquifex aeolicus,
68 Thermotoga maritima,
69 Thermus thermophilus,
70 Deinococcus radiodurans,
71 Dehalococcoides ethenogenes,
72 Nostoc sp. PCC 7120,
73 Synechocystis sp. PCC6803,
74 Synechococcus elongatus,
75 Synechococcus sp. WH8102,
76 Prochlorococcus marinus MIT9313,
77 Prochlorococcus marinus SS120,
78 Prochlorococcus marinus CCMP1378,
79 Gloeobacter violaceus,
80 Gemmata obscuriglobus,
81 Rhodopirellula baltica,
82 Leptospira interrogans L1-130,
83 Leptospira interrogans 56601,
84 Treponema pallidum,
85 Treponema denticola,
86 Borrelia burgdorferi,
87 Tropheryma whipplei TW08/27,
88 Tropheryma whipplei Twist,
89 Bifidobacterium longum,
90 Corynebacterium glutamicum 13032,
91 Corynebacterium glutamicum,
92 Corynebacterium efficiens,
93 Corynebacterium diphtheriae,
94 Mycobacterium bovis,
95 Mycobacterium tuberculosis CDC1551,
96 Mycobacterium tuberculosis H37Rv,
97 Mycobacterium leprae,
98 Mycobacterium paratuberculosis,

99 Streptomyces avermitilis,
100 Streptomyces coelicolor,
101 Fibrobacter succinogenes,
102 Chlorobium tepidum,
103 Porphyromonas gingivalis,
104 Bacteroides thetaiotaomicron,
105 Chlamydophila pneumoniae TW183,
106 Chlamydia pneumoniae J138,
107 Chlamydia pneumoniae CWL029,
108 Chlamydia pneumoniae AR39,
109 Chlamydophila caviae,
110 Chlamydia muridarum,
111 Chlamydia trachomatis,
112 Thermoanaerobacter tengcongensis,
113 Clostridium tetani,
114 Clostridium perfringens,
115 Clostridium acetobutylicum,
116 Mycoplasma mobile,
117 Mycoplasma pulmonis,
118 Mycoplasma pneumoniae,
119 Mycoplasma genitalium,
120 Mycoplasma gallisepticum,
121 Mycoplasma penetrans,
122 Ureaplasma parvum,
123 Mycoplasma mycoides,
124 Phytoplasma Onion yellows,
125 Listeria monocytogenes F2365,
126 Listeria monocytogenes EGD,
127 Listeria innocua,
128 Oceanobacillus iheyensis,
129 Bacillus halodurans,
130 Bacillus cereus ATCC 14579,
131 Bacillus cereus ATCC 10987,
132 Bacillus anthracis,
133 Bacillus subtilis,
134 Staphylococcus aureus MW2,
135 Staphylococcus aureus N315,
136 Staphylococcus aureus Mu50,

137 Staphylococcus epidermidis,
138 Streptococcus agalactiae III,
139 Streptococcus agalactiae V,
140 Streptococcus pyogenes M1,
141 Streptococcus pyogenes MGAS8232,
142 Streptococcus pyogenes MGAS315,
143 Streptococcus pyogenes SSI-1,
144 Streptococcus mutans,
145 Streptococcus pneumoniae R6,
146 Streptococcus pneumoniae TIGR4,
147 Lactococcus lactis,
148 Enterococcus faecalis,
149 Lactobacillus johnsonii,
150 Lactobacillus plantarum,
151 Thalassiosira pseudonana,
152 Cryptosporidium hominis,
153 Plasmodium falciparum,
154 Oryza sativa,
155 Arabidopsis thaliana,
156 Cyanidioschyzon merolae,
157 Dictyostelium discoideum,
158 Eremothecium gossypii,
159 Saccharomyces cerevisiae,
160 Schizosaccharomyces pombe,
161 Anopheles gambiae,
162 Drosophila melanogaster,
163 Takifugu rubripes,
164 Danio rerio,
165 Rattus norvegicus,
166 Mus musculus,
167 Homo sapiens,
168 Pan troglodytes,
169 Gallus gallus,
170 Caenorhabditis elegans,
171 Caenorhabditis briggsae,
172 Leishmania major,
173 Giardia lamblia,
174 Nanoarchaeum equitans,

```
175 Sulfolobus tokodaii,
176 Sulfolobus solfataricus,
177 Aeropyrum pernix,
178 Pyrobaculum aerophilum,
179 Thermoplasma volcanium,
180 Thermoplasma acidophilum,
181 Methanobacterium thermautotrophicum,
182 Methanopyrus kandleri,
183 Methanococcus maripaludis,
184 Methanococcus jannaschii,
185 Pyrococcus horikoshii,
186 Pyrococcus abyssi,
187 Pyrococcus furiosus,
188 Archaeoglobus fulgidus,
189 Halobacterium sp. NRC-1,
190 Methanosarcina acetivorans,
191 Methanosarcina mazei
;
 tree iTOL_exported_tree = [&R] (((((((((((((((((((1:0.00000,2:0.00000):0.00044[9
End;
```

Genome Table

```
NCBI_ID Scientific_Name
240015 Acidobacterium capsulatum ATCC 51196
56636 Aeropyrum pernix
63363 Aquifex aeolicus
2234 Archaeoglobus fulgidus
198094 Bacillus anthracis str. Ames
222523 Bacillus cereus ATCC 10987
226900 Bacillus cereus ATCC 14579
86665 Bacillus halodurans
1423 Bacillus subtilis
818 Bacteroides thetaiotaomicron
959 Bdellovibrio bacteriovorus
216816 Bifidobacterium longum
518 Bordetella bronchiseptica
519 Bordetella parapertussis
```

520 Bordetella pertussis
139 Borrelia burgdorferi
375 Bradyrhizobium japonicum
118099 Buchnera aphidicola (Acyrthosiphon pisum)
135842 Buchnera aphidicola (Baizongia pistaciae)
98794 Buchnera aphidicola (Schizaphis graminum)
6238 Caenorhabditis briggsae
197 Campylobacter jejuni
203907 Candidatus Blochmannia floridanus
155892 Caulobacter vibrioides
83560 Chlamydia muridarum
813 Chlamydia trachomatis
83557 Chlamydophila caviae
115711 Chlamydophila pneumoniae AR39
115713 Chlamydophila pneumoniae CWL029
182082 Chlamydophila pneumoniae TW-183
1097 Chlorobaculum tepidum
536 Chromobacterium violaceum
1488 Clostridium acetobutylicum
1502 Clostridium perfringens
1513 Clostridium tetani
1717 Corynebacterium diphtheriae
152794 Corynebacterium efficiens
196627 Corynebacterium glutamicum ATCC 13032
1718 Corynebacterium glutamicum
777 Coxiella burnetii
45157 Cyanidioschyzon merolae
243164 Dehalococcoides ethenogenes 195
882 Desulfovibrio vulgaris subsp. vulgaris str. Hildenborough
1351 Enterococcus faecalis
562 Escherichia coli
155864 Escherichia coli O157:H7 EDL933
83334 Escherichia coli O157:H7
59374 Fibrobacter succinogenes subsp. succinogenes S85
76856 Fusobacterium nucleatum subsp. nucleatum
35554 Geobacter sulfurreducens
33072 Gloeobacter violaceus
730 Haemophilus ducreyi

727 Haemophilus influenzae
32025 Helicobacter hepaticus
210 Helicobacter pylori
85963 Helicobacter pylori J99
33959 Lactobacillus johnsonii
1590 Lactobacillus plantarum
1360 Lactococcus lactis subsp. lactis
5664 Leishmania major
1642 Listeria innocua
1639 Listeria monocytogenes
265669 Listeria monocytogenes str. 4b F2365
381 Mesorhizobium loti
2190 Methanocaldococcus jannaschii
39152 Methanococcus maripaludis
2320 Methanopyrus kandleri
2214 Methanosarcina acetivorans
2209 Methanosarcina mazei
187420 Methanothermobacter thermautotrophicus str. Delta H
1770 Mycobacterium avium subsp. paratuberculosis
1765 Mycobacterium bovis
1769 Mycobacterium leprae
83331 Mycobacterium tuberculosis CDC1551
83332 Mycobacterium tuberculosis H37Rv
2096 Mycoplasma gallisepticum
2097 Mycoplasma genitalium
267748 Mycoplasma mobile 163K
44101 Mycoplasma mycoides subsp. mycoides SC
28227 Mycoplasma penetrans
2104 Mycoplasma pneumoniae
2107 Mycoplasma pulmonis
160232 Nanoarchaeum equitans
65699 Neisseria meningitidis serogroup A
491 Neisseria meningitidis serogroup B
915 Nitrosomonas europaea
182710 Oceanobacillus iheyensis
100379 Onion yellows phytoplasma
747 Pasteurella multocida
141679 Photorhabdus luminescens subsp. laumondii

837 Porphyromonas gingivalis
1219 Prochlorococcus marinus
74547 Prochlorococcus marinus str. MIT 9313
59919 Prochlorococcus marinus subsp. pastoris str. CCMP1986
287 Pseudomonas aeruginosa
160488 Pseudomonas putida KT2440
323 Pseudomonas syringae pv. tomato
13773 Pyrobaculum aerophilum
29292 Pyrococcus abyssi
2261 Pyrococcus furiosus
53953 Pyrococcus horikoshii
305 Ralstonia solanacearum
1076 Rhodopseudomonas palustris
781 Rickettsia conorii
782 Rickettsia prowazekii
209261 Salmonella enterica subsp. enterica serovar Typhi Ty2
601 Salmonella typhi
70863 Shewanella oneidensis
198215 Shigella flexneri 2a str. 2457T
623 Shigella flexneri
382 Sinorhizobium meliloti
234267 Solibacter usitatus Ellin6076
158878 Staphylococcus aureus subsp. aureus Mu50
196620 Staphylococcus aureus subsp. aureus MW2
158879 Staphylococcus aureus subsp. aureus N315
1282 Staphylococcus epidermidis
216495 Streptococcus agalactiae serogroup III
216466 Streptococcus agalactiae serogroup V
1309 Streptococcus mutans
1313 Streptococcus pneumoniae
171101 Streptococcus pneumoniae R6
1314 Streptococcus pyogenes
198466 Streptococcus pyogenes MGAS315
186103 Streptococcus pyogenes MGAS8232
193567 Streptococcus pyogenes SSI-1
33903 Streptomyces avermitilis
1902 Streptomyces coelicolor
2287 Sulfolobus solfataricus

```
111955 Sulfolobus tokodaii
32046 Synechococcus elongatus
84588 Synechococcus sp. WH 8102
1148 Synechocystis sp. PCC 6803
119072 Thermoanaerobacter tengcongensis
2303 Thermoplasma acidophilum
50339 Thermoplasma volcanium
2336 Thermotoga maritima
262724 Thermus thermophilus HB27
158 Treponema denticola
160 Treponema pallidum
203267 Tropheryma whipplei str. Twist
218496 Tropheryma whipplei TW08/27
134821 Ureaplasma parvum
666 Vibrio cholerae
36870 Wigglesworthia glossinidia endosymbiont of Glossina brevipalpis
66077 Wolbachia sp. wMel
844 Wolinella succinogenes
92829 Xanthomonas axonopodis pv. citri
340 Xanthomonas campestris pv. campestris
2371 Xylella fastidiosa
183190 Xylella fastidiosa Temecula1
632 Yersinia pestis
187410 Yersinia pestis KIM
```

4.2 Parameters

The varying genome coverage samples were created with CAMISIM, with config-files as well as genomes and all output provided upon request, with XX being the desired coverage:
`./metagenomesimulation configXX.ini`
The genome similarity samples were also created with CAMISIM and the provided config-files, mapping files and genomes, where XX is a number corresponding to one of the 152 iTol genomes:
`./metagenomesimulation configXX.ini`
The iTol genomes were evolved manually using the sgEvolver using a specific template tree available on request:
`./sgEvolver template.tree genome.fa genome.fa evolved_genome.bam`

evolved_genome.fa
MEGAHIT 1.0.3/1.12 was run with default parameters on all the read sets:
```
./megahit --12 anonymous_reads.fq -o Assembly/
```
SPAdes was called with the `--meta` flag (from here on called metaSPAdes) and lstinline!–phred-offset! parameter set to 33 in case quality information on the reads was missing (and on 48 cores with `-t 48`):
```
./spades.py --12 anonymous_reads.fq --meta -t 48 --phred-offset
33 -o metaSPAdes
```
quast 3.1 was run for the coverage experiment:
`./quast.py -R e_coli_k12.fa -o quast contigs.fasta` (for the E.coli coverage sweep)
For the similarity experiment, metaquast was run with a small patch (now built in into newer version of quast) to allow mapping to only a single reference genome for multiple best hits ("strain contigs") or to multiple reference genomes ("consensus contigs") using the ambiguity-usage flag of the nucmer mapping.
```
./metaquast.py -R genome_ancestor.fa,genome_evolved.fa -o quast
contigs.fasta
./metaquast.py.OLD -R genome_ancestor.fa,genome_evolved.fa -o quast
 contigs.fasta
```
Where the latter is the unpatched evaluating consensus contigs and the first the patched evaluating strain contigs.
The data sets (mousegut and HMP) where created using the from_profile script of CAMISIM versions 0.2, 0.2.1 and 0.2.2 respectively:
```
./metagenome_from_profile -p profile.biom -o out
```
All scripts for creating tables, plots, config files are also available, either upon request or already under `data.cami-challenge.org/participate`.

4.3 PICRUST

We created functional profiles with PICRUSt, using a prediction model from 3772 KEGG genomes and corressponding 16S rRNA sequences according the PICRUSt "Genome Prediction Tutorial": 1) The determination of 16S rRNA copy numbers was performed by rrnDB Estimate version 5.2.2) KEGG Orthology (KO) profiles of the genomes were extracted fromthe KEGG database Release 77. 3) A tree was created from GreenGenes reference alignment (Version 13.5 OTU-RepSet 97) and the 16S rRNA of the KEGG genomes to build a integrated prediction model.

4.4 Config file

Due to its flexibility, CAMISIM requires an extensive configuration file. A default config for both short and long reads is shipped within CAMISIM and is exemplary shown here:

```
 [Main]
# maximum number of processes
max_processors=8

# 0: community design + read simulator,
# 1: read simulator only
phase=0

# ouput directory, where the output will be stored (will be
   overwritten if set in from_profile)
output_directory=

# temporary directory
temp_directory=/tmp

# gold standard assembly
gsa=True

# gold standard for all samples combined
pooled_gsa=True

# anonymize sequences?
anonymous=True

# id of dataset, used in foldernames and is prefix in anonymous
    sequences
dataset_id=RL

# Read Simulation settings, relevant also for from_profile
[ReadSimulator]
# which readsimulator to use:
# Choice of 'art', 'wgsim', 'nanosim', 'pbsim'
```

```
type=art

# Samtools (http://www.htslib.org/) takes care of sam/bam files
   . Version 1.0 or higher required!
# file path to executable
samtools=tools/samtools-1.3/samtools

# file path to read simulation executable
readsim=tools/art_illumina-2.3.6/art_illumina

#error profiles:
#for ART:
#HiSeq 150bp: hi150
#MBARC-26 150bp: mbarc
#for wgsim:
#error rate as <float> (e.g. 0.05 for 5% error rate)
#blank for nanosim and wgsim
profile=mbarc

# Directory containing error profiles (can be blank for wgsim)
error_profiles=tools/art_illumina-2.3.6/profiles/

#paired end read, insert size (not applicable for nanosim)
fragments_size_mean=270
fragment_size_standard_deviation=27

# Everything in this section is only required for de novo
   simulation:
[CommunityDesign]
# specify the samples size in Giga base pairs
size=5

# how many different samples?
number_of_samples=1

# how many communities
num_communities=1
```

```
# directory containing the taxdump of ncbi, version from
   22.02.2017 is shipped
# "nodes.dmp"
# "merged.dmp"
# "names.dmp"
ncbi_taxdump=tools/ncbi-taxonomy_20170222.tar.gz

# the strain simulator for de novo strain creation
strain_simulation_template=scripts/StrainSimulationWrapper/
   sgEvolver/simulation_dir/

# define communities: [community<integer>]
[community0]
# information about all included genomes:
# can be used for multiple samples
metadata=
id_to_genome_file=

# how many genomes do you want to sample overall?
genomes_total=
num_real_genomes=

# how many genomes per species taxon
# (species taxon will be replaced by OTU-cluster later on)
max_strains_per_otu=1
ratio=1

# which kind of different samples do you need?
# replicates / timeseries_lognormal / timeseries_normal /
   differential
mode=

# Part: community design
# Set parameters of log-normal and normal distribution, number
   of samples
# sigma > 0; influences shape (higher sigma -> smaller peak and
    longer tail),
log_sigma=2
```

```
# mu (real number) is a parameter for the log-scale
log_mu=1

# do you want to see a distribution before you decide to use it
   ? yes/no
view=no

# second community
#[community1]
#metadata=defaults/plasmid_metadata_v2.3_filtered.tsv
#id_to_genome_file=defaults/plasmid_path.tsv
#genomes_total=20
#max_strains_per_otu=3
#ratio=2.5
#mode=differential
#log_mu=1
#log_sigma=2
#view=false
```

5 OTU mapping file

The mouse gut BIOM file and thus dataset includes metagenomic data from different mice, vendors and sites in the gut. The exact assignment of each sample in the data set to the mice/vendors (as a code) and location in the gut is found in the following mapping file:

```
biom16S-SampleID SampleID-CAMISIM Microbiota Location
500.L21 0 HZI Ileum
502.L21 1 HZI Colon
503.L21 2 N6 Ileum
504.L21 3 N6 Cecum
506.L21 4 Jan-10C Ileum
507.L21 5 Jan-10C Cecum
508.L21 6 Jan-10C Colon
509.L21 7 NCI Ileum
```

510.L21 8 NCI Cecum
511.L21 9 NCI Colon
512.L21 10 Tac-401 Ileum
513.L21 11 Tac-401 Cecum
514.L21 12 Tac-401 Colon
515.L21 13 Tac-401 Ileum
516.L21 14 Tac-401 Cecum
517.L21 15 Tac-401 Colon
518.L21 16 Tac-130 Ileum
520.L21 17 Tac-130 Colon
521.L21 18 Tac-130 Ileum
522.L21 19 Tac-130 Cecum
523.L21 20 Tac-130 Colon
524.L21 21 ChR-9 Ileum
526.L21 22 ChR-9 Colon
527.L21 23 ChR-9 Ileum
528.L21 24 ChR-9 Cecum
529.L21 25 ChR-9 Colon
530.L21 26 ChR-11 Ileum
531.L21 27 ChR-11 Cecum
532.L21 28 ChR-11 Colon
533.L21 29 ChR-11 Ileum
534.L21 30 ChR-11 Cecum
535.L21 31 ChR-11 Colon
536.L21 32 Har-2 Ileum
537.L21 33 Har-2 Cecum
538.L21 34 Har-2 Colon
539.L21 35 Har-2 Ileum
540.L21 36 Har-2 Cecum
541.L21 37 Har-2 Colon
542.L21 38 Jan-1A Ileum
543.L21 39 Jan-1A Cecum
544.L21 40 Jan-1A Colon
545.L21 41 Jan-1A Ileum
546.L21 42 Jan-1A Cecum
547.L21 43 Jan-1A Colon
548.L21 44 ChR-7 Cecum
549.L21 45 Tac-809 Cecum

550.L21 46 N6 Content
551.L21 47 NCI Content
552.L21 48 Jan-10C Content
553.L21 49 HZI Content
554.L21 50 N6 Content
555.L21 51 Jan-10C Content
556.L21 52 NCI Content
557.L21 53 Tac-809 DistalColon
559.L21 54 Tac-809 ProximalColon
560.L21 55 Tac-809 DistalColon
561.L21 56 Tac-809 ProximalColon
562.L21 57 Tac-809 Cecum
563.L21 58 ChR-7 DistalColon
564.L21 59 ChR-7 ProximalColon
565.L21 60 ChR-7 DistalColon
566.L21 61 ChR-7 ProximalColon
567.L21 62 ChR-7 Cecum
569.L21 63 ChR-7 Ileum
570.L21 64 Prevotella Culture
